# Investigating the Anti-inflammatory Mechanism of *Alpinia purpurata* (Vieill.) K. Schum. Rhizome Extract: Metabolite Profiling, Network Pharmacology, in vitro Safety, and in vivo Validation

**DOI:** 10.5812/ijpr-169725

**Published:** 2026-05-26

**Authors:** Kiki Mulkiya Yuliawati, Fahriza Salsabilla Yuniar, Ellin Febrina, Gofarana Wilar, Raden Maya Febriyanti, Deshanda Kurniawan Prayoga, Faisal Kuswandani, Sri Adi Sumiwi, Supat Jiranusornkul, Jutti Levita

**Affiliations:** 1 Faculty of Pharmacy, Universitas Padjadjaran, Jatinangor, Indonesia; 2 Faculty of Mathematics and Natural Sciences, Bandung Islamic University, Bandung, Indonesia; 3 Department of Pharmacology and Clinical Pharmacy, Faculty of Pharmacy, Universitas Padjadjaran, Jatinangor, Indonesia; 4 Department of Biological Pharmacy, Faculty of Pharmacy, Universitas Padjadjaran, Jatinangor, Indonesia; 5 Department of Oral Biology, Faculty of Dentistry, Universitas Padjadjaran, Bandung, Indonesia; 6 Department of Pharmaceutical Science, Faculty of Pharmacy, Chiang Mai University, Chiang Mai, Thailand

**Keywords:** *Alpinia* Species, Drug Discovery, Inflammation, Quercetin, Shogaol, Zingiberaceae

## Abstract

**Background:**

*Alpinia purpurata* has shown promising effects in alleviating inflammatory conditions; however, its underlying mechanisms require further investigation.

**Objectives:**

This study aimed to elucidate the molecular mechanisms underlying the anti-inflammatory activity of *A. purpurata* rhizome extract (EEAP) and its active metabolites through the TLR4/MyD88 pathway.

**Methods:**

The EEAP was analyzed for proximate composition, vitamin C content, total phenolic content (TPC), total flavonoid content (TFC), total monomeric anthocyanin content (TMAC), and metabolite profile, followed by a network pharmacology analysis. Cytotoxicity was evaluated in HEK-293 cells. An in vivo study of carrageenan-induced paw edema in rats was conducted to validate the findings.

**Results:**

EEAP contained 42.12% ash, 34.66% moisture, 6.03% protein, 16.60% fat, and 0.59% carbohydrate. The vitamin C content was 941.55 mg/100 g extract, the TPC was 452.9 mg GAE/100 g extract, the TFC was 416.1 mg QUE/100 g extract, and the TMAC was 2770 mg/100 g extract. Functional enrichment analysis identified the TLR4/MyD88 signaling pathway as contributing to IL-23 production. Eight metabolites with anti-inflammatory properties were verified based on their Pa values: glycidyl oleate, 1-stearoylglycerol, alpha-linolenic acid, methyl palmitate, shogaol, 4-methoxybenzaldehyde, ginkgoneolic acid, and curcumene. EEAP was not toxic to HEK-293 cells (IC_50_= 741.1 μg/mL) compared with quercetin (IC_50_= 96.73 μg/mL) and cisplatin (IC_50_= 7.44 μg/mL). EEAP reduced edema volume but did not alter TLR4 or MyD88 expression under the present experimental conditions.

**Conclusions:**

EEAP is not toxic to normal cells and exerts an anti-inflammatory effect by reducing edema volume in carrageenan-induced rats; however, it does not alter the TLR4/MyD88 pathway.

## 1. Background

Plant-derived polyphenols, flavonoids, and other secondary metabolites are actively characterized and studied for their anti-inflammatory properties (1). *Alpinia* spp. (Zingiberaceae) are used as spices and in folkloric medicine for the treatment of inflammatory disorders, but rigorous experimental substantiation remains limited (2). Reported activities include antioxidant effects, suppression of pro-inflammatory cytokines, improvements in gastrointestinal disorders, and enhanced ulcer healing (3).

Among these species, *A. purpurata* is particularly notable. An ethnobotanical survey in South Jakarta found that it had the highest utility value and fidelity level, indicating broad recognition and consistent use for specific ailments (4). However, its bioactive constituents and mechanisms remain incompletely defined. GC-MS profiling has identified 42 essential-oil components in Brazilian *A. purpurata* flowers, dominated by alpha-pinene, beta-pinene, and beta-caryophyllene (5); 13 compounds in flowers from Tamil Nadu (6); and a fatty alcohol compound in n-hexane leaf extracts (7). Rhizome extracts contain alpha-pinene, beta-pinene, 1,8-cineole, (E)-methyl cinnamate, and multiple gingerols/shogaols (2).

A few studies have reported the anti-inflammatory and immunomodulatory activities of *A. purpurata* (8). In one study, lectin from the inflorescences induced the release of Th1 (T-helper cell type 1) and Th17 cytokines and activated IL-10 production (8). Intracellular inflammatory pathways are activated by toll-like receptors (TLRs) through interactions between their TIR domains and adaptor proteins, such as myeloid differentiation factor 88 (MyD88) (9).

## 2. Objectives

The present study aimed to elucidate the molecular mechanisms underlying the anti-inflammatory activity of *A. purpurata* rhizome extract (EEAP) and its active metabolites via the TLR4/MyD88 pathway by integrating metabolite profiling, phytochemical analysis, network pharmacology, cytotoxicity testing, and in vivo assessment in carrageenan-induced rat models. The primary outcomes were edema volume and TLR4 and MyD88 expression.

## 3. Methods

### 3.1. Plant Collection and Identification

Whole plants and rhizomes of red galangal were collected in Lembang, West Java, Indonesia, in March 2024. Botanical identification was performed by Arifin Surya Dwipa Irsyam (Scopus ID: 57211286941), a certified botanist at the School of Life Sciences and Technology, Bandung Institute of Technology, Indonesia. The material was authenticated as *Alpinia purpurata* (Vieill.) K. Schum. (Zingiberaceae), and a voucher specimen was deposited (No. 2063/2024).

### 3.2. Extract Preparation

Approximately 10.84 kg of fresh rhizomes were washed using standard pharmacognosy laboratory procedures, chopped, and air-dried for 6 days, yielding 900 g of dried material. The dried rhizomes were milled to obtain 840.21 g of coarse powder. Maceration was performed with 70% ethanol for 3 × 24 hours at 26 ± 2°C, with continuous stirring during the first 6 hours of each cycle and solvent replacement every 24 hours. The combined filtrates were filtered and concentrated under reduced pressure using a rotary evaporator (Büchi R-300) at 50°C and 70 rpm, yielding 61.11 g of ethanolic extract (EEAP).

### 3.3. Proximate and Vitamin C Analyses

Proximate and nutritional analyses of EEAP were performed at the Test Service Laboratory, Faculty of Agricultural Technology, *Universitas Padjadjaran*. All analyses followed Indonesian National Standard SNI 01-2891-1992 and AOAC Official Methods of Analysis (2023). Carbohydrate content was calculated by subtracting the sum of moisture, ash, protein, and fat from 100 g of sample.

### 3.4. Total Phenolic Content, Total Flavonoid Content, and Total Monomeric Anthocyanin Content

The TPC of EEAP was determined using the Folin-Ciocalteu method (The Ministry of Health of the Republic of Indonesia, 2017). EEAP (0.2 g) was dissolved in methanol (25 mL) and stirred for 30 minutes. An aliquot (1 mL) of EEAP was mixed with 7.5% (w/v) Folin-Ciocalteu reagent, incubated for 5 minutes at room temperature, and then combined with 10% (w/v) sodium carbonate; absorbance was measured at 730 nm. TPC was calculated from a gallic acid calibration curve (20 - 40 μg/mL; y = 0.0151x - 0.0034, R^2^ = 0.9942) and expressed as mg gallic acid equivalents (GAE)/100 g dry weight of EEAP.

TFC was determined using the aluminum chloride (AlCl_3_) colorimetric assay (The Ministry of Health of the Republic of Indonesia, 2017). EEAP (0.2 g) was prepared in ethanol (25 mL). An aliquot (0.5 mL) was reacted with 10% (w/v) AlCl_3_ in ethanol and 1 M sodium acetate (0.1 mL), incubated for 30 minutes at room temperature, and measured at 440 nm. The TFC was derived from a quercetin standard curve (20 - 60 μg/mL; y = 0.008x - 0.013, R^2^ = 0.9987) and expressed as mg quercetin equivalents (QUE)/100 g dry weight of EEAP.

TMAC was determined using the pH differential method (10-12). The EEAP (250 mg) was dissolved in distilled water (50 mL) and separately diluted with 0.025 M KCl buffer (pH 1.0) and 0.4 M sodium acetate buffer (pH 4.5). After 30 minutes at room temperature, samples were centrifuged (7000 rpm, 15 minutes, 4°C), and supernatant absorbance was recorded at 520 and 700 nm.

### 3.5. Metabolite Profiling by Ultrahigh-Performance Liquid Chromatography-High-Resolution Double Tandem Mass Spectrometry

Metabolites in EEAP were analyzed using an ultrahigh-performance liquid chromatography-high-resolution double tandem mass spectrometry (UHPLC-HRMS/MS) system. Chromatographic separation was performed on a Thermo Scientific Vanquish UHPLC Binary Pump equipped with an Accucore Phenyl-Hexyl column (100 mm × 2.1 mm, 2.6 μm) maintained at 40°C. The mobile phases were MS-grade water with 0.1% formic acid (A) and MS-grade methanol with 0.1% formic acid (B), delivered at 0.3 mL/min under gradient elution: 5% B to 90% B over 16 minutes, held at 90% B for 4 minutes, and then re-equilibrated to 5% B for 25 minutes. Data were acquired in full-scan mode with data-dependent MS/MS for structural annotation. Metabolites were tentatively identified by matching spectra to online libraries, including MzCloud (https://www.mzcloud.org/), ChemSpider (https://www.chemspider.com/), and PubChem (https://pubchem.ncbi.nlm.nih.gov/).

### 3.6. Network Pharmacology

#### 3.6.1. Gene-Disease Association

Target genes were selected based on their association with the disease of interest using databases such as GeneCards (https://www.genecards.org/), a general gene-centric database, or DisGeNET (http://www.disgenet.org/), a highly specialized database with highly curated gene-disease associations (GDAs) (13).

In this study, GDAs for inflammation were retrieved from DisGeNET by querying “inflammation” using default parameters. Genes linked to inflammation were compiled together with the disease specificity index (DSI), disease pleiotropy index (DPI), and gene-disease association score (GDAS) (14).

#### 3.6.2. Protein-Protein Interaction Network

Protein-protein interactions involving TLR4 and MYD88 were constructed using STRING (15), with a confidence threshold of 0.7, which is expected to represent a high-confidence cutoff for including interactions in the analysis. TLR4 and MYD88 were queried with *Homo sapiens* specified as the organism, and the resulting interaction network was visualized as nodes (proteins) and edges (interactions).

#### 3.6.3. Gene-Disease Expression Enrichment

The Enrichr library (https://maayanlab.cloud/Enrichr/) was used to evaluate enrichment of inflammation-related diseases and associated gene-expression signatures for the target proteins and their co-expressed genes, supporting the mechanistic interpretation of inflammatory pathways. Default parameters were used as follows: organism, human; number of terms returned, 50; and input format, gene symbols in a plain-text list (16). WikiPathways 2024 Human and KEGG 2021 Human enrichment analyses were used in this study to obtain a comprehensive list of target genes and their associated biological functions.

#### 3.6.4. In Silico Bioactivity Prediction

Putative anti-inflammatory activity of the 30 most abundant EEAP metabolites was predicted using PASS Online (Way2Drug). SMILES strings were submitted for each metabolite, and predictions were interpreted using Pa (probability of activity) values (17). Activities with Pa > 0.7 were considered highly likely to be experimentally confirmed, whereas Pa < 0.5 indicated a low likelihood of activity (18). In this study, only the 30 most abundant metabolites were further analyzed in the network pharmacology analysis. Studies often integrate metabolomics with network pharmacology to prioritize metabolites based on abundance or relevance. For example, a recent study on stress-relieving plants identified 100 metabolites but focused only on prominent ones for protein targets and pathways (19). Another study of Dendrobium officinale identified 628 metabolites but selected only 29 differential metabolites with potential pharmacological activity for network pharmacology analysis (20).

### 3.7. Cytotoxicity Assay in HEK-293 Cells

HEK-293 cells (ATCC CRL-1573; Pharmacy Translational Research Laboratory collection, Faculty of Pharmacy, *Universitas Padjadjaran*) were maintained at 37°C in a humidified 5% CO_2_ incubator in DMEM supplemented with 10% fetal bovine serum and 1% penicillin-streptomycin (Gibco 670087). At approximately 80% confluence, cells were washed twice with PBS, detached with 0.25% trypsin-0.53 mM EDTA, and used for cytotoxicity testing.

The EEAP cytotoxicity was assessed using a Cell Counting Kit-8 (CCK-8; WST-8, CK04 - 11, Dojindo Europe) assay. A stock solution was prepared by dissolving EEAP in 1% DMSO in DMEM and serially diluting it to obtain final concentrations of 7.812 - 1000 μg/mL. Quercetin and cisplatin were evaluated as comparator compounds using the same assay workflow. The vehicle control consisted of 1% DMSO in culture medium without the test extract. IC_50_ values were calculated using GraphPad Prism 10.

### 3.8. Effects of EEAP on Carrageenan-Induced Edema in Male Wistar Rats

#### 3.8.1. Animal Handling, Allocation, and Ethical Approval

The study was not prospectively preregistered in a public registry but was conducted under institutional ethics approval. All animal procedures were approved by the Research Ethics Committee of *Universitas Padjadjaran* (approval No. 1165/UN6.KEP/EC/2024; approved November 7, 2024), complied with the Guide for the Care and Use of Laboratory Animals (8th ed.; NRC, 2011), and followed ARRIVE 2.0 reporting guidelines. A total of 45 male Wistar rats (6 - 8 weeks old; 200 ± 20 g) were obtained from the Animal Breeding Facility, Division of Animal Laboratories, PT Biofarma (West Java, Indonesia), and housed at 24 ± 2°C and 55% relative humidity under a 12-hour light/dark photoperiod in cages (52 × 45 × 45 cm) with wood-husk bedding, which was replaced every 3 days. The animals received a standard diet (Prospets; 10 g/rat/day) and water ad libitum throughout a 5-day acclimatization period and the in vivo study at the Animal Research and Innovation Center, Faculty of Pharmacy, *Universitas Padjadjaran*. All procedures were conducted under the supervision of Dr. Aziiz Mardanarian Rosdianto (Scopus ID: 57212213886). Animals were monitored daily. Key humane endpoint criteria included > 20% body weight loss, abnormal behavior, loss of appetite, and lack of grooming. During acclimatization, no morbidity or mortality occurred; therefore, all animals were included in the analyses.

After acclimatization, the rats were assigned by simple random allocation, with no formal assignment method, to 15 experimental groups (n = 3 per group), representing 5 treatment conditions at 3 terminal time points (T_0_, T_100_, and T_200_). The use of n = 3 per group was based on sample-size prediction using G*Power Statistical Power Analyses, set to one-way ANOVA, fixed-model, alpha of 0.05, power of 0.95, and an effect size f^2^ of 0.30 (medium), resulting in a total sample size of 38; however, 45 rats were used. No formal allocation concealment procedure was used after randomization. Blinding was not implemented during treatment administration, plethysmometric measurement, tissue processing, or Western blot densitometric analysis. According to the predefined experimental schedule, animals received 1% Na-CMC, diclofenac sodium, or EEAP based on group allocation, followed 30 minutes later by carrageenan induction in all groups except the normal control group. Paw edema volume was measured at the assigned terminal time point, after which the animals were euthanized, and left hind paw tissue was excised for Western blot analysis of TLR4 and MyD88. The overall structure of the animal experiment is summarized in Table 1.

**Table 1.** Summary of Animal Grouping, Interventions, Terminal Time Points, and Collected Endpoints ^a^

Treatment Condition	Carrageenan Induction	Dose/Treatment	T_0_ (n)	T_100_ (n)	T_200_ (n)	Total (n)
**Control**						
Normal	No	1% Na-CMC	3	3	3	9
Negative	Yes	1% Na-CMC	3	3	3	9
Positive	Yes	Diclofenac sodium 1 mg/kg dispersed in 1% Na-CMC	3	3	3	9
**EEAP**						
Low dose	Yes	EEAP 250 mg/kg BW dispersed in 1% Na-CMC	3	3	3	9
High dose	Yes	EEAP 500 mg/kg BW dispersed in 1% Na-CMC	3	3	3	9
**Total**	-	-	15	15	15	45

^a^ T_0_, T_100_, and T_200_ indicate terminal sampling time points at 0, 100, and 200 minutes, respectively. Separate groups were used at each time point because the animals were euthanized immediately after paw edema measurement to collect their left hind paw tissue for Western blot analysis of TLR4 and MyD88. Abbreviations: Na-CMC, sodium carboxymethyl cellulose; EEAP, ethanolic extract of *A. purpurata* rhizome.

#### 3.8.2. Anti-Inflammatory Assay: Carrageenan-Induced Paw Edema

After acclimatization, rats were stratified and allocated into 15 groups (n = 3 per group), representing 5 treatment conditions assessed at 3 terminal time points (T_0_, T1_00_, and T_200_). Diclofenac sodium, EEAP, or 1% Na-CMC was administered 30 minutes before edema induction by subplantar injection of 1% carrageenan into the left hind paw, except in the normal control groups, which did not receive carrageenan.

Paw edema volume was measured using a plethysmometer at the assigned terminal time point: immediately before induction (T_0_), 100 minutes after carrageenan injection (T_100_), or 200 minutes after carrageenan injection (T_200_). Immediately after measurement, rats were euthanized in a sealed chamber using 5% isoflurane in oxygen for 60 seconds by trained personnel. Death was confirmed by the absence of respiration for 1 minute, no heartbeat, and no ocular reflex. The left hind paw tissue was then excised and snap-frozen in liquid nitrogen for protein analysis.

The terminal time points of 0, 100, and 200 minutes after carrageenan injection were selected because edema fluid accumulation, which is related to the known kinetics of carrageenan-induced inflammation, is biphasic. The first phase begins immediately after irritant injection and diminishes within 60 minutes. The second period of accelerated edema formation begins at 60 minutes, peaks at approximately 120 minutes, and persists through 180 minutes. The first phase accounts for approximately 40% of the total edema volume produced over 180 minutes, with the period from 120 to 180 minutes representing the maximum edema peak (21).

#### 3.8.3. Protein Extraction

Frozen paw tissue was minced, lysed in RIPA buffer (Sigma-Aldrich, R0278), and centrifuged at 15,000 rpm for 5 minutes. The lysate supernatant (15 μL) was mixed 1:1 with sample buffer and heat-denatured at 95°C for 5 minutes.

#### 3.8.4. Western Blotting of TLR4 and MyD88

In the Western blot analysis, paw tissue from rats in the same group was pooled. Proteins were separated on a 15% separating gel using sodium dodecyl sulfate-polyacrylamide gel electrophoresis, prepared by combining acrylamide, 1.5 M Tris-HCl pH 8.8, 10% SDS, ammonium persulfate, and TEMED (Sigma-Aldrich; catalog number not available), and transferred onto a nitrocellulose/PVDF membrane (Thermo Fisher Scientific; catalog number not available) in a Mini Gel Tank (Thermo Fisher Scientific), assembled as cathode-sponge-filter paper-gel-membrane-filter paper-sponge-anode (12 V, 200 mA, 30 minutes). Membranes were stained with Ponceau S for 5 minutes, washed with phosphate-buffered saline with Tween-20, and blocked with 3% bovine serum albumin. Membranes were incubated overnight at 4°C with primary antibodies against TLR4 (mouse monoclonal; FineTest; catalog number FNab09837) or MyD88 (KO-validated rabbit monoclonal; ABclonal; catalog number A19082), washed, and incubated with an anti-mouse secondary antibody for 90 minutes at room temperature, followed by PBS washes. Bands were visualized using a Li-Cor Odyssey CLX imaging system. Expected band sizes were approximately 100 kDa for TLR4, approximately 33 kDa for MyD88, and approximately 45 kDa for beta-actin. Beta-actin (Santa Cruz Biotechnology; catalog number sc-47778) was used as the housekeeping protein/loading control to normalize protein bands. Band intensities were quantified by densitometry. The ladder used was the Bio-Helix Blu11 Prestained Protein Ladder (catalog number PMB11 - 0500). Western blot analysis was performed as a single measurement.

## 4. Results

### 4.1. Proximate Composition and Vitamin C

The proximate and nutritional composition of EEAP comprised 42.12% ash, 34.66% moisture, 6.03% protein, 16.60% fat, 0.59% carbohydrate, and 941.55 mg/100 g vitamin C.

### 4.2. Total Phenolic Content, Total Flavonoid Content, and Total Monomeric Anthocyanin Content

The TPC, calculated using the gallic acid calibration curve, was 452.9 mg GAE/100 g extract. The TFC, calculated using the quercetin calibration curve, was 416.1 mg QUE/100 g extract. TMAC, determined using the pH differential method, was 2770 mg/100 g extract.

### 4.3. Metabolite Profiling by UHPLC-HRMS/MS

**Figure 1.**
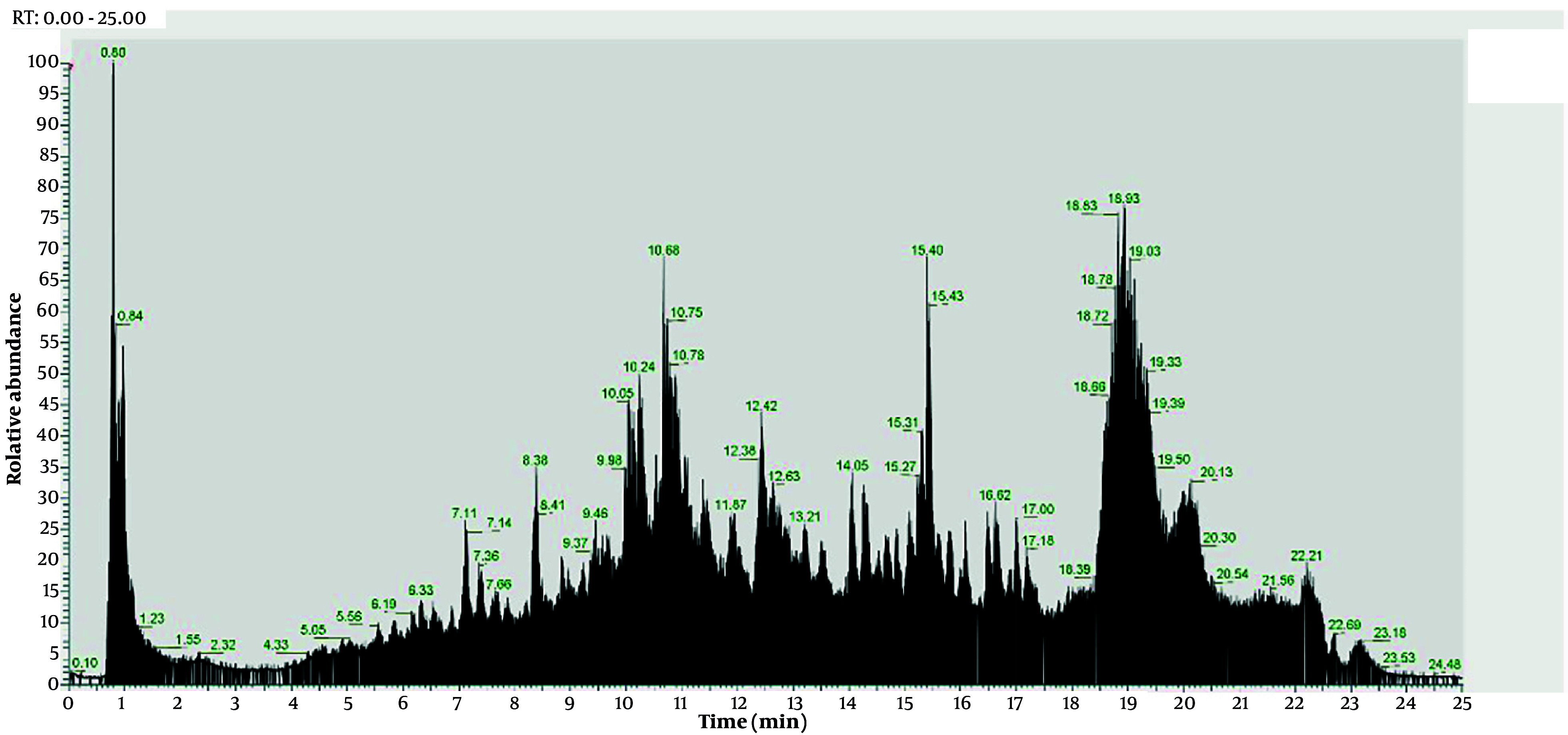
UHPLC chromatogram of EEAP 1 mg in 1 mL of 96% methanol at 40°C. The mobile phase consisted of mass spectrometry-grade water with 0.1% formic acid and mass spectrometry-grade methanol with 0.1% formic acid. The gradient program was initially set at 5% B, gradually increased to 90% B within 16 minutes, held at 90% for 4 minutes, and then returned to the initial condition (5% B) for 25 minutes. The flow rate was set at 0.3 mL/min, with an injection volume of 3 μL.

UHPLC-HRMS/MS data were annotated using MzCloud, ChemSpider, and PubChem and included 22 compounds previously reported to have anti-inflammatory activity (Table 2). Representative chromatograms are shown in Figure 1. The complete UHPLC-HRMS/MS dataset, including molecular formulas, accurate masses, and MS/MS fragmentation patterns, is provided in the Table S1 in Supplementary File. In this study, the metabolite standards initiative classification was not assigned because chemoinformatic tools, such as Van Krevelen diagrams and DBE calculations, and automated workflows, such as MetExtract and XCMS, were not available in the laboratory.

**Table 2.** UHPLC-HRMS/MS Data of Metabolites in the Ethanolic Extract of Alpinia Purpurata Rhizome ^a^

Name of Compound [Class of Metabolite]	Retention Time (min)	Relative Abundance (%)
**Beauvericin**	15.409	42.40
**1-Stearoyl glycerol**	15.303	7.12
**9(Z),11(E),13(E)-Octadecatrienoic acid methyl ester**	16.285	3.80
**Alpha-linolenic acid**	14.326	2.56
**4-oxo-4,5,6,7-tetrahydrobenzo[b]furan-3-carboxylic acid**	4.428	2.36
**Cinnamic acid**	6.188	2.30
**(E,E)-alpha-Farnesene**	13.118	1.97
**Monoolein**	12.861	1.55
**Alpha-eleostearic acid**	12.452	1.19
**Ginkgoneolic acid**	10.332	1.15
**Methyl palmitate**	17.071	1.05
**Hirsuteine [monoterpene indole alkaloid]**	5.956	0.86
**Sitostenone**	18.918	0.82
**Fagaramide**	2.797	0.74
**Homovanillic acid**	4.737	0.70
**Glycidyl oleate**	15.832	0.65
**Curcumene**	9.763	0.67
**Lachnophyllum ester**	10.043	0.59
**Limonin**	7.317	0.57
**6-Pentyl-2H-pyran-2-one**	9.686	0.56
**Methyl isonicotinate**	0.805	0.50
**Shogaol**	11.893	0.49
**4-Methoxybenzaldehyde**	10.025	0.47
**Lotaustralin**	0.797	0.47
**4-Coumaric acid**	5.203	0.47
**Helenalin**	5.835	0.43
**2-Amino-1,3,4-octadecanetriol**	9.924	0.42
**Butyrin**	13.2	0.42
**Coumarin**	7.701	0.38
**(-)-Caryophyllene oxide**	9.104	0.37
**Ethylestrenol**	12.717	0.37
**Cholest-4-en-3-one**	18.151	0.35
**Prolylleucine**	1.159	0.34
**Campest-4-en-3-one**	18.499	0.33
**Eugenitin**	5.835	0.32
**Hernanol**	10.296	0.32
**Acridine-9(10H)-thione**	11.728	0.31
**2-[(1S)-1-Hydroxyethyl]-4(1H)-quinazolinone**	4.308	0.30
**2-{2-[5-(Ethoxycarbonyl)-2-morpholinoanilino]-2-oxoethoxy} acetic acid**	9.122	0.29
**Piperine**	10.753	0.26
**(4-benzyl-1,4-oxazinan-2-yl) methyl N-[(4-methylphenyl) sulfonyl] carbamate**	13.227	0.26
**Phytosphingosine**	11.949	0.25
**Methyl cinnamate**	7.685	0.25

^a^ All metabolites were putatively annotated using UHPLC-HRMS/MS chromatograms and spectral matching against public databases; no authentic reference standards were used for confirmation.

### 4.4. Network Pharmacology

DisGeNET analysis indicated that, under inflammatory conditions, TLR4 had a DSI of 0.31, DPI of 0.96, and GDAS of 0.95. In addition, under inflammatory conditions, MYD88 had a DSI of 0.40, a DPI of 0.91, and a GDAS of 0.75.

In DisGeNET, the DSI ranges from 0.23 to 1.0. A low DSI indicates that a gene is linked to a broader range of diseases, whereas a high DPI indicates a wider range of disease classes, reflecting greater pleiotropy (https://blog.disgenet.com/disease-specificity-index-dsi-disease-pleiotropy-index-dpi/). In this study, the TLR4 and MYD88 genes, with low DSI and high DPI, were highly associated with a broader range of inflammation classes. This suggests that these genes are critical, multifunctional nodes in human disease networks.

**Figure 2.**
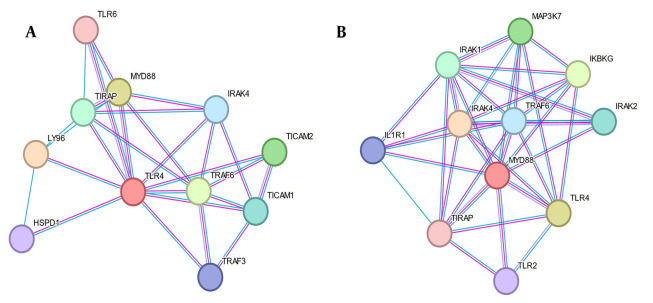
Protein-protein interaction network diagram of A, TLR4 and B, MYD88 generated using the STRING database.

STRING-derived protein-protein interaction networks identified first-neighbor interactors of TLR4 as MYD88, TLR6, TIRAP, LY96, HSPD1, IRAK4, TRAF3, TRAF6, TICAM1, and TICAM2 (Figure 2A). First-neighbor interactors of MYD88 were TLR4, TLR2, TIRAP, IRAK2, IKBKG, TRAF6, IRAK4, IL1R1, IRAK1, and MAP3K7 (Figure 2B). TIRAP, IRAK4, and TRAF6 were shared first-neighbor proteins for both TLR4 and MYD88 (Figure 2).

**Figure 3.**
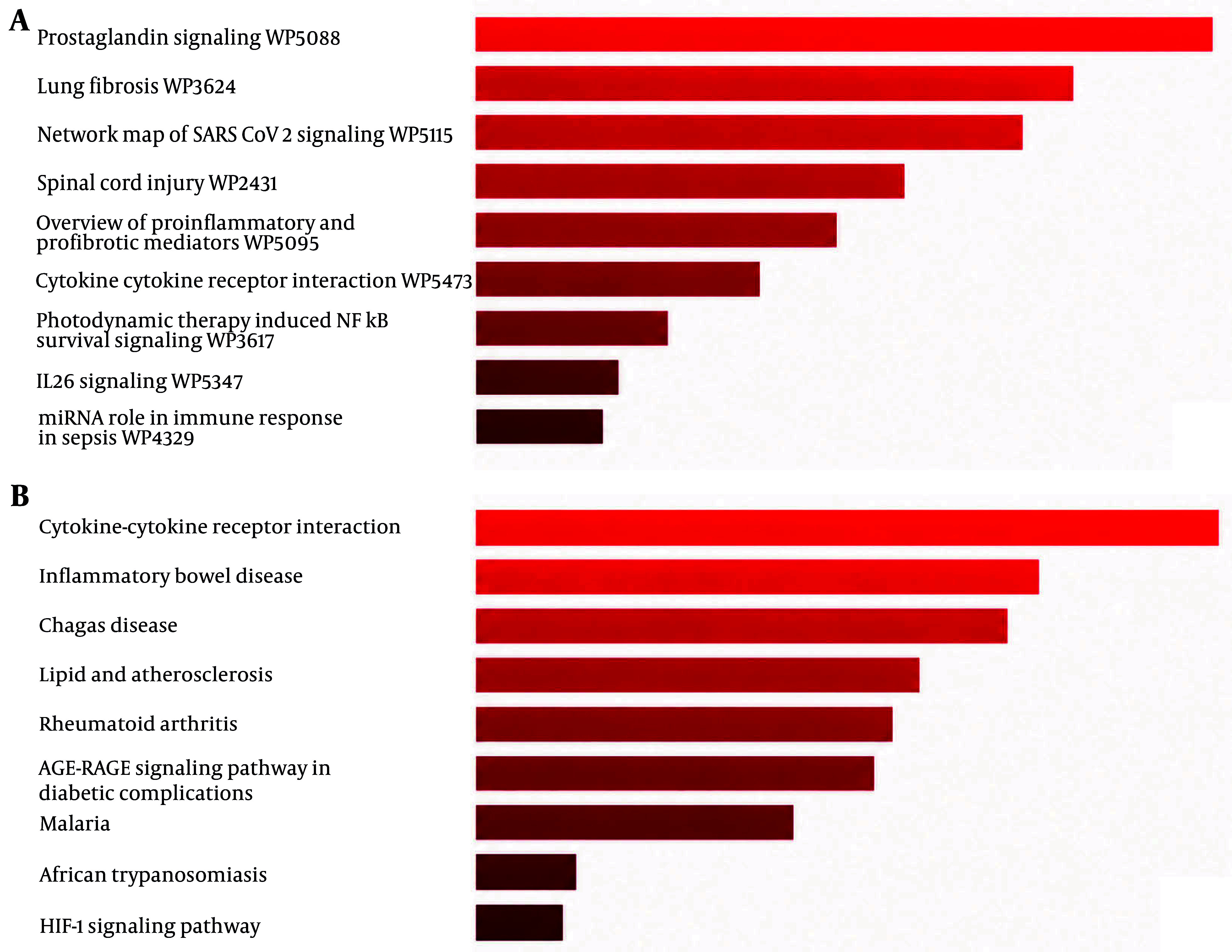
Bar plots obtained from A, WikiPathways 2024 Human and B, KEGG 2021 Human databases via Enrichr for inflammatory conditions.

Enrichr analysis summarized inflammation-associated pathologies linked to the TLR4/MYD88-centered gene set, depicted in a bar plot (Figure 3), using the WikiPathways 2024 Human and Kyoto Encyclopedia of Genes and Genomes (KEGG) 2021 Human gene sets. The analysis comprised 136 inflammation-relevant genes. WikiPathways 2024 Human enrichment implicated prostaglandin signaling, lung fibrosis, SARS-CoV-2 signaling network maps, spinal cord injury, pro-inflammatory/pro-fibrotic mediators, cytokine-cytokine receptor interaction, inflammatory bowel disease signaling, photodynamic therapy-induced NF-kappaB survival signaling, IL-2 signaling, and mRNA-mediated immune responses in sepsis (Figure 3A). In addition, KEGG 2021 Human enrichment highlighted cytokine-cytokine receptor interaction, inflammatory bowel disease, Chagas disease, lipid and atherosclerosis, rheumatoid arthritis, IL-17 signaling, AGE/RAGE signaling in diabetic complications, malaria, African trypanosomiasis, and HIF-1 signaling (Figure 3B).

PASS Online prediction of the 30 most abundant EEAP metabolites identified those with Pa > 0.7 for anti-inflammatory activity: glycidyl oleate (0.877), 1-stearoylglycerol (0.809), alpha-linolenic acid (0.804), methyl palmitate (0.758), shogaol (0.717), 4-methoxybenzaldehyde (0.712), ginkgoneolic acid (0.706), and curcumene (0.700) (Table 3).

**Table 3.** PASS Prediction of the Probability of Ethanolic Extract of A. Purpurata Rhizome Metabolites Being Active for Anti-Inflammatory Activity ^a^

Metabolite	Pa (Probability of Activity)	Predicted Activity ^b^
**Glycidyl oleate**	0.877 ^c^	CYP2J substrate
**1-Stearoylglycerol**	0.809 ^c^	CYP2J substrate
**Alpha-linolenic acid**	0.804 ^c^	Anti-inflammatory
**4-oxo-4,5,6,7-tetrahydrobenzo[b]furan-3-carboxylic acid**	0.800 ^c^	CYP2J substrate
**Methyl palmitate**	0.758 ^c^	Anti-inflammatory, intestinal
**Shogaol**	0.717 ^c^	Anti-inflammatory
**4-Methoxybenzaldehyde**	0.712 ^c^	CYP2J substrate
**Ginkgoneolic acid**	0.706 ^c^	Anti-inflammatory, intestinal
**Curcumene**	0.700 ^c^	Anti-inflammatory
**Monoolein**	0.689	Anti-inflammatory
**Sitostenone**	0.682	Anti-inflammatory, ophthalmic
**Alpha-eleostearic acid**	0.675	Anti-inflammatory
**(E,E)-alpha-Farnesene**	0.669	Anti-inflammatory
**Cinnamic acid**	0.656	Anti-inflammatory
**Homovanillic acid**	0.633	Anti-inflammatory
**Lachnophyllum ester**	0.620	Anti-inflammatory, intestinal
**Lotaustralin**	0.610	Anti-inflammatory
**Methyl isonicotinate**	0.597	CYP2J substrate
**Fagaramide**	0.575	TNF expression inhibitor
**6-Pentyl-2H-pyran-2-one**	0.570	Anti-inflammatory

^a^ Abbreviations: TNF, tumor necrosis factor.

^b^ CYP2J substrate is a catalyst of arachidonic acid metabolism.

^c^ Pa values indicated a strong probability of the metabolite being active for anti-inflammatory activity (Pa ≥ 0.7).

### 4.5. Cytotoxicity Assay in HEK-293 Cells

**Figure 4.**
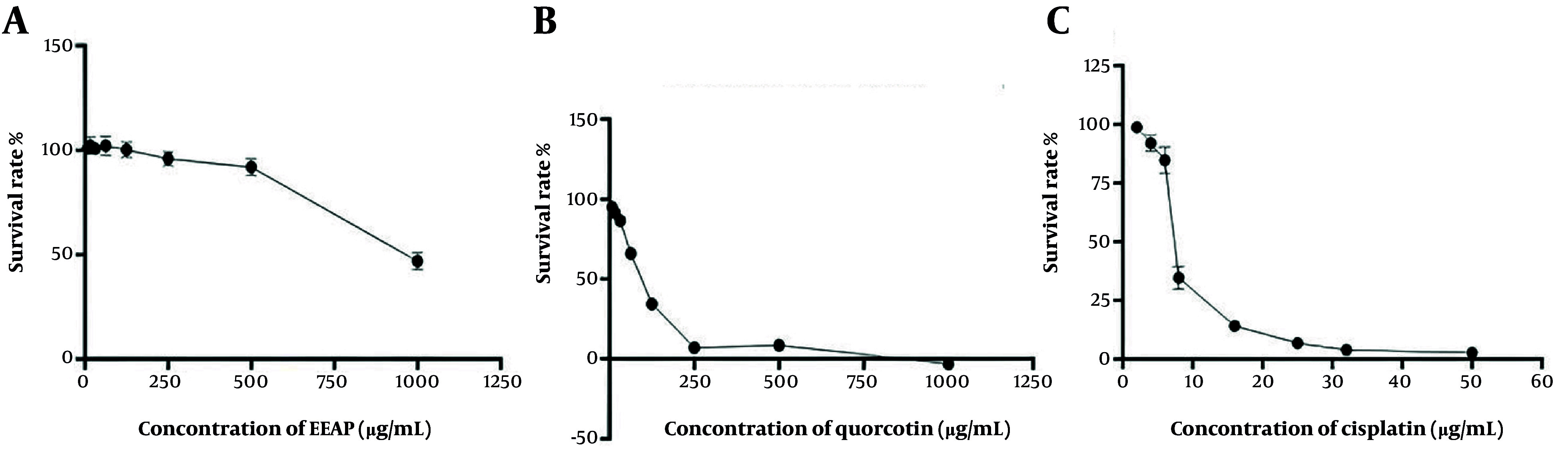
Cytotoxicity assay of (A) EEAP (IC50= 741.1 μg/mL), (B) quercetin (IC50 = 96.73 μg/mL), and (c) cisplatin (IC50 = 7.44 μg/mL).

In the HEK-293 cytotoxicity assay, EEAP showed low cytotoxicity (IC_50_ = 741.1 μg/mL) compared with quercetin (IC_50_ = 96.73 μg/mL) and cisplatin (IC_50_ = 7.44 μg/mL). These data indicate low cytotoxicity in this non-immune cell model under basal culture conditions (Figure 4).

### 4.6. Effects of EEAP on Edema Volume in Carrageenan-Induced Edema in Male Wistar Rats

The network pharmacology results were validated by evaluating the effects of EEAP in carrageenan-induced edema in male Wistar rats. Edema volume, one of the primary outcomes, is presented in Table 4.

**Table 4.** Effects of Ethanolic Extract of A. Purpurata Rhizome on Carrageenan-Induced Paw Edema in Male Wistar Rats ^a, b^

Groups	Edema Volume Increase (mL) at T_100_	Edema Volume Increase (mL) at T_200_
**Normal control (Na-CMC 1%)**	0.00 ± 0.00	0.00 ± 0.00
**Negative control (Na-CMC 1%)**	0.20 ± 0.00	0.17 ± 0.00
**Positive control (Na diclofenac in Na-CMC 1%)**	0.073 ± 0.023 (P = 0.047) ^c^	0.060 ± 0.000 (P = 0.041) ^c^
**EEAP 250 mg/kg BW in Na-CMC 1%**	0.073 ± 0.023 (P = 0.047) ^c^	0.073 ± 0.023 (P = 0.047) ^c^
**EEAP 500 mg/kg BW in Na-CMC 1%**	0.073 ± 0.023 (P = 0.047) ^c^	0.063 ± 0.029 (P = 0.041) ^c^

^a^ Values are expressed as mean ± SD (n = 3 rats/group).

^b^ Significant differences between groups were analyzed using one-way analysis of variance followed by Tukey test.

^c^ Significant difference compared with the negative control group (P < 0.05).

At T_100_, representing 100 minutes after carrageenan injection, increased paw edema volume was observed in all groups except the normal control group. The negative control group exhibited the greatest increase in edema volume (0.20 ± 0.00 mL). In contrast, the positive control and EEAP-treated groups (250 and 500 mg/kg body weight) showed significantly lower edema volume (0.073 ± 0.023 mL) than the negative control group (P < 0.05), indicating the anti-inflammatory activity of EEAP. The 95% confidence intervals for the treatment groups were relatively wide (0.016 - 0.130), reflecting variability and the small sample size.

At T_200_, representing 200 minutes after carrageenan injection, reduced paw edema volume was observed in the positive control and EEAP-treated groups. The positive control and EEAP 500 mg/kg body weight groups showed edema volume increases of 0.060 ± 0.000 mL and 0.063 ± 0.029 mL, respectively, which were significantly lower than that of the negative control group (P < 0.05). In contrast, the EEAP 250 mg/kg body weight group did not show a further reduction in edema volume and remained at 0.073 ± 0.023 mL. At this time point, however, substantial overlap of confidence intervals was noted among the treatment groups. Although the treatment groups demonstrated reduced edema volume compared with the negative control group, the overlapping confidence intervals indicate that the differences may not be statistically robust. This is likely due to the small sample size (n = 3 per group), which limits statistical power and increases uncertainty.

### 4.7. Western Blot Analysis of TLR4 and MyD88 in the Left Hind Paw

**Figure 5.**
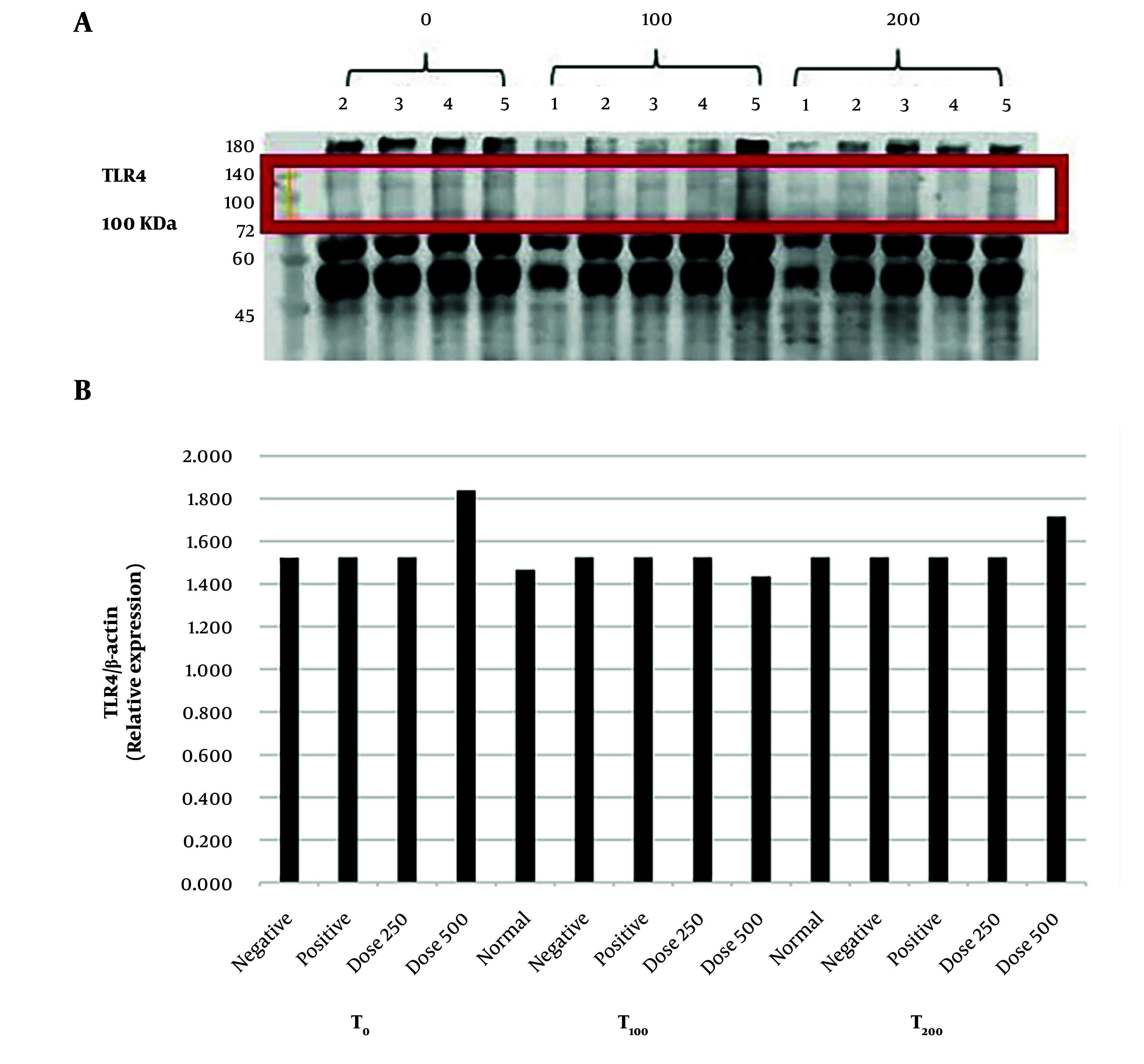
Effects of EEAP on TLR4 expression in the left hind paw of carrageenan-induced Wistar rats based on a single Western blot measurement. (A) TLR4 protein bands with a molecular weight of 100 kDa on the nitrocellulose membrane. (B) TLR4/beta-actin relative expression calculated using a densitometric technique. No statistical test was performed because the Western blot was conducted as a single measurement.

**Figure 6.**
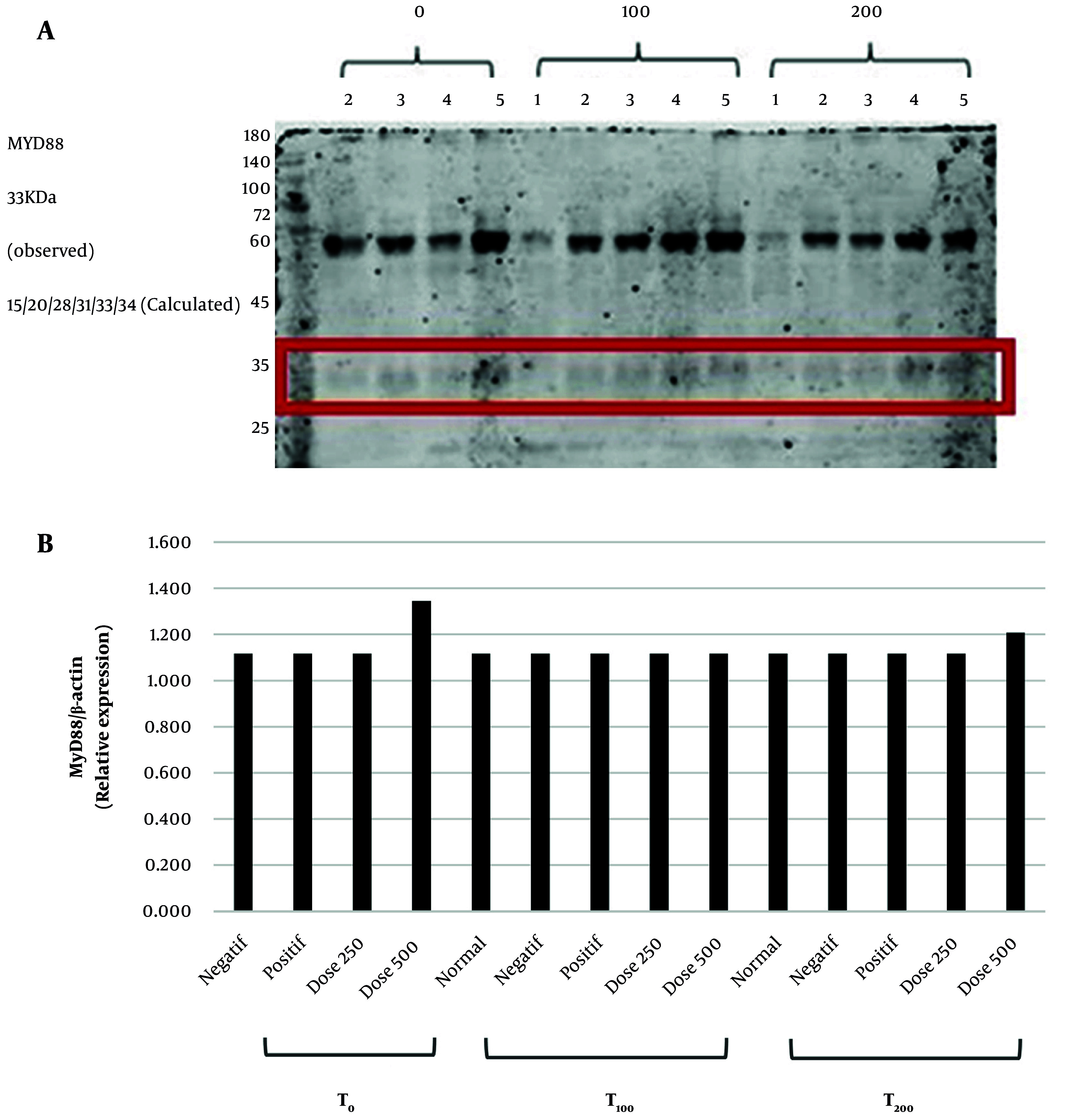
Effects of EEAP on MyD88 expression in the left hind paw of carrageenan-induced Wistar rats based on a single Western blot measurement. (A) MyD88 protein bands with an observed molecular weight of 33 kDa on the nitrocellulose membrane. (B) MyD88/beta-actin relative expression calculated using a densitometric technique. No statistical test was performed because the Western blot was conducted as a single measurement.

Western blot analysis showed that EEAP did not alter TLR4 (Figure 5) or MyD88 (Figure 6) expression under the present experimental conditions. Although EEAP significantly reduced carrageenan-induced paw edema, this effect was not accompanied by modulation of total TLR4 or MyD88 protein expression in paw tissue.

## 5. Discussion

*A. purpurata* remains of pharmacological interest, and in this study, we characterized an ethanol extract of rhizomes cultivated in Lembang, West Java, Indonesia (EEAP). EEAP showed a proximate profile of 42.12% ash, 34.66% moisture, 6.03% protein, 16.60% fat, and 0.59% carbohydrate, with vitamin C at 941.55 mg/100 g. TPC was 452.9 mg GAE/100 g, TFC was 416.1 mg QUE/100 g, TMAC was 2770 mg/100 g, and UHPLC-HRMS/MS annotated 62 metabolites (Table 2), dominated by beauvericin. This observation should be interpreted cautiously because beauvericin is a mycotoxin typically associated with fungal secondary metabolism rather than a well-established endogenous metabolite of *A. purpurata*. Its apparent predominance may therefore reflect contamination, post-harvest fungal growth, or annotation uncertainty arising from database-based spectral matching in the absence of authentic standards.

TPC and TFC were lower than the values reported for *A. purpurata* from Jember (TPC 1350 mg GAE/100 g; TFC 1780 mg QUE/100 g) (22) and Malang (TPC 14876 mg GAE/100 g; TFC 3105 mg QUE/100 g) (23). Phytochemical profiling identified 62 rhizome metabolites (Table 1), extending prior GC-MS reports for *A. purpurata* flowers and leaves from Brazil (42 essential-oil constituents) (5) and flowers from Tamil Nadu (13 metabolites) (6).

DisGeNET analysis in the network pharmacology study indicated that, under inflammatory conditions, the DSI of TLR4 was 0.31, DPI was 0.96, and GDAS was 0.95. Meanwhile, the DSI of MYD88 was 0.40, DPI was 0.91, and GDAS was 0.75. This was considered low specificity, meaning that the test has a high false-positive rate (for a DSI of 0.31, 69% of non-diseased individuals are incorrectly classified as diseased, while for a DSI of 0.40, 60% of non-diseased individuals are incorrectly classified as diseased) (24). DPI and GDAS are metrics used in clinical research to evaluate the complexity and strength of gene–disease relationships. A pleiotropy index of 0.96 (TLR4) and 0.91 (MyD88) indicates high pleiotropy, meaning that the gene is strongly implicated in multiple diseases or phenotypes. This aligns with findings that pleiotropic genes often play essential roles in biological systems and are enriched in deleterious variants (25). High pleiotropy suggests that the gene may be central to shared biological pathways across diseases, making it a potential target for therapeutic interventions (26). GDAS measures the strength and confidence of the association between a gene and a specific disease based on aggregated evidence from genetic studies. A GDAS of > 0.75 reflects a strong association between the gene and the disease (27).

Network pharmacology and enrichment analyses identified TLR4/MyD88-associated signaling as a plausible inflammation-related pathway potentially relevant to the activity of EEAP. These analyses were used as hypothesis-generating tools to identify candidate targets and interacting proteins, including TIRAP, IRAK4, and TRAF6. Diclofenac was included in this study as a reference anti-inflammatory drug in the carrageenan-induced edema model. Because diclofenac primarily exerts anti-inflammatory effects through cyclooxygenase inhibition, it should not be interpreted as a mechanistic comparator for TLR4/MyD88 signaling.

To our knowledge, network pharmacology has not been applied to *A. purpurata*. Related studies include *A. officinarum* (28-31), *A. katsumadai* (32), and *A. oxyphyllae* (33). Across these reports, PI3K-Akt signaling recurrently emerged as a central pathway, consistent with Alpinia phytochemicals acting on growth, survival, and inflammatory nodes.

TLR4/MyD88 signaling drives transcriptional programs for pro-inflammatory cytokines and type I interferons (34, 35). MyD88 is a core adaptor in MyD88-dependent signaling, coupling TLR4 via TIR-domain interactions and engaging IRAK4 through its death domain (33). Consistently, IRAK4 appeared among the shared first-neighbor interactors of TLR4 and MyD88 in our protein–protein interaction analysis. TLR4 activation can also rapidly engage PI3K, promoting downstream Akt phosphorylation (36), providing a plausible interface between innate immune activation and PI3K-Akt signaling.

PASS prediction identified 8 abundant EEAP metabolites with Pa > 0.7 for anti-inflammatory activity (Table 2). Glycidyl oleate, an oleic acid ester reported in virgin olive oil, has documented anti-inflammatory effects (37) and was predicted to interact with multiple inflammatory targets, including TLR4, in a study of *Liriope platyphylla* seed constituents (38). TLR4 is activated by pathogen-associated molecular patterns and pro-inflammatory stimuli such as carrageenan and LPS, initiating NF-kappaB signaling and downstream pro-inflammatory gene expression (39). Evidence for 1-stearoylglycerol is limited, but cassava leaf-derived 1-stearoylglycerol inhibited NF-kappaB signaling and reduced TNF-alpha, IL-1beta, and IL-6 (40). Alpha-linolenic acid suppresses inflammatory mediators and NF-kappaB activation in LPS-stimulated macrophages (41), is associated with reduced CRP and improved lipid profiles in humans (42), protects against LPS-induced acute lung injury (43), and selectively inhibits COX-2 in rodent inflammatory models induced by carrageenan (1). Methyl palmitate reduced carrageenan-induced edema and decreased PGE_2_, TNF-alpha, and IL-6 (44), with additional cardioprotective anti-inflammatory activity reported in myocardial injury models (45). Shogaols have repeatedly been shown to modulate inflammatory cascades, particularly through NF-kappaB regulation and NLRP3 inflammasome control (46-50).

Safety profiling of *A. purpurata* extracts is scarce and largely limited to cancer cell lines such as OAW42 and HeLa (47, 51). EEAP did not show cytotoxicity in normal HEK-293 cells compared with quercetin and cisplatin. HEK-293 cells, derived from human embryonic kidney tissue, were used as a preliminary screen of general cellular tolerability in a non-immune mammalian cell line. These cells are used in biomedical research because of their ease of cultivation, high transfection efficiency, and adaptability to various experimental conditions (52). Although these cells are not immune cells, they offer several advantages as a safety model for evaluating anti-inflammatory agents and inflammation-related pathways, such as the NF-kappaB pathway (53, 54). HEK-293 cells transfected with TLR2 and TLR4 have shown inflammatory responses induced by lipoteichoic acid and LPS. These pathways involve downstream signaling molecules such as MyD88 and NF-kappaB, which regulate the production of TNF-alpha and IL-6 (55).

In vivo evidence for *A. purpurata* in carrageenan-induced edema has not been previously reported. In our model, EEAP reduced carrageenan-induced inflammation in male Wistar rats, although inhibition of TLR4 and MyD88 expression was modest. Consistent with the anti-inflammatory potential across the genus, volatile oil from *A. calcarata* and its major compounds significantly reduced ear thickness, weight, myeloperoxidase activity, and cytokine levels in the formalin-induced pain model in mice (56).

Taken together, these findings support a two-level interpretation. First, the in silico analyses generated a mechanistic hypothesis implicating TLR4/MyD88-associated inflammatory signaling. Second, the in vivo experiments confirmed that EEAP exerted anti-inflammatory activity but did not validate significant modulation of TLR4 or MyD88 protein expression under the conditions tested. These results may be related to the small sample size (n = 3 per group) and the single Western blot measurement, which are limitations of this study. Therefore, the TLR4/MyD88 pathway should be regarded as a plausible but unconfirmed mechanistic hypothesis requiring further validation using larger sample sizes and broader molecular readouts.

### 5.1. Strengths and Limitations of the Study

This study combines metabolite profiling, network pharmacology, cytotoxicity screening, and in vivo anti-inflammatory testing to examine the potential activity of EEAP. Nevertheless, several limitations should be considered. The in vivo experiment used a small group size (n = 3 per group), limiting statistical power and increasing susceptibility to biological and technical variability, particularly for a single Western blot densitometric analysis. As a result, the absence of significant changes in TLR4 and MyD88 should be interpreted cautiously. In addition, the proposed mechanistic involvement of the TLR4/MyD88 axis was derived primarily from in silico predictions and was not confirmed experimentally, because Western blot analysis showed no significant modulation of either protein. The metabolite assignments were also putative because they were based on database-assisted UHPLC-HRMS/MS annotation without confirmation by authentic standards. This limitation is particularly relevant for beauvericin, a mycotoxin annotated as the dominant metabolite, because its apparent prominence raises safety and analytical validity concerns that require targeted confirmation. Therefore, the present findings support anti-inflammatory potential at the phenotypic level but do not establish a definitive mechanism or safety. Further studies with larger sample sizes, targeted metabolite validation, and expanded mechanistic assays are needed.

### 5.2. Conclusions

This study characterized the nutritional composition, metabolite profile, predicted molecular mechanisms, and cytotoxicity of an ethanol extract of *A. purpurata* (Vieill.) K. Schum. rhizome (EEAP). EEAP contained 42.12% ash, 34.66% moisture, 6.03% protein, 16.60% fat, 0.59% carbohydrate, vitamin C 941.55 mg/100 g, TPC 452.9 mg GAE/100 g, TFC 416.1 mg QUE/100 g, and TAC 2770 mg/100 g extract. UHPLC-HRMS/MS annotated 62 metabolites. Network analysis identified TIRAP, IRAK4, and TRAF6 as shared first-neighbor interactors of TLR4 and MyD88, and enrichment implicated TLR4/MyD88 signaling in IL-23 production. PASS prediction highlighted 8 metabolites with Pa > 0.7 for anti-inflammatory activity (glycidyl oleate, 1-stearoylglycerol, alpha-linolenic acid, methyl palmitate, shogaol, 4-methoxybenzaldehyde, ginkgoneolic acid, and curcumene), but these constituents were present at low relative abundance. EEAP showed low cytotoxicity in HEK-293 cells under the assay conditions used and significantly reduced carrageenan-induced paw edema, with no alteration in TLR4 and MyD88 expression.

ijpr-25-1-169725-s001.pdf

## Data Availability

The dataset presented in the study is available on request from the corresponding author during submission or after publication. The data are not publicly available due to ethical and confidentiality considerations.
