# Supplementary material for: Investigating the Anti-inflammatory Mechanism of Alpinia purpurata (Vieill.) K. Schum. Rhizome Extract: Metabolite Profiling, Network Pharmacology, in vitro Safety, and in vivo Validation
Source: Iran J Pharm Res. 2026 May 26;25(1):e169725. doi: 10.5812/ijpr-169725 (PMC13401191; doi:10.5812/ijpr-169725)
Supplement: ijpr-25-1-169725-s001.pdf [file ijpr-25-1-169725-s001.pdf]

Table S1. UHPLC-HRMS/MS data of metabolites in the ethanolic extract of *Alpinia purpurata* (Vieill.) K. Schum rhizome

**Name of Compound [Class of Metabolite]**	**Molecular Formula**	**[M+H] + m/z**		**Retention Time (min)**	**Main Product Ions (MS/MS, m/z)**	**Area (Max)**	**Relative Abundance (%)**
		**Calculated Mass (Da)**	**Accurate Mass (Da)**				
Beauvericin [Trimeric cyclohexadepsipeptide mycotoxin]	$C_{45}H_{57}N_3O_9$ [PubChem CID 3007984]	783.408	784.416	15.409	785.418, 793.389, 807.399, 823.373	19.558 x 10^8	42.40
1-Stearoylglycerol [Fatty acid ester]	$C_{21}H_{42}O_4$ [PubChem CID 22035687]	358.307	359.314	15.303	342.306, 358.302, 360.317, 361.320, 379.283, 380.286	3.285 x 10^8	7.12
9(Z),11(E),13(E)-Octadecatrienoic Acid methyl ester [Fatty acid methyl ester]	$C_{19}H_{32}O_2$ [PubChem CID 11277787]	292.239	293.247	16.285	292.102, 292.141, 294.250, 295.189, 295.253, 297.241, 296.255,	1.755 x 10^8	3.80
α-Linolenic acid [Omega (ω)-3 fatty acid]	$C_{18}H_{30}O_2$ [PubChem CID 5280934]	278.224	279.231	14.326	278.182, 280.234, 281.237, 281.247 282.278, 283.204	1.183 x 10^8	2.56
4-oxo-4,5,6,7-tetrahydrobenzo[b]furan-3-carboxylic acid [Benzofuran]	$C_9H_8O_4$ [PubChem CID 713937]	180.042	181.049	4.428	179.991, 181.103, 182.052, 182.984, 183.053, 183.064, 184.096, 184.985	1.090 x 10^8	2.36
Cinnamic acid [Aromatic carboxylic acid]	$C_9H_8O_2$ [PubChem CID 444539]	148.052	149.059	6.188	149.024, 149.094, 149.131, 150.062, 152.106, 153.054, 153.127	1.062 x 10^8	2.30
(E,E)-α-Farnesene [Sesquiterpene]	$C_{15}H_{24}$ [PubChem CID 5281516]	204.187	205.195	13.118	205.086, 206.198, 207.174, 207.201, 209.096, 209.153	0.908 x 10^8	1.97
Monoolein [Monoglyceride]	$C_{21}H_{40}O_4$ [PubChem CID 5283468]	356.291	357.299	12.861	340.291, 345.202, 353.265, 355.223	0.713 x 10^8	1.55
(2E)-2-(hydroxymethyl)-3-[3-oxo-5-(propan-2-yl)-1,3,4,5,6,7-hexahydro-2-benzofuran-4-yl]prop-2-enoic acid [Terpene lactone]	$C_{15}H_{20}O_5$ [PubChem CID 45782757]	280.131	281.138	7.681	281.117, 293.171, 302.564, 305.098	0.677 x 10^8	1.47

NP-006370 [Natural product]	$C_{15}H_{22}O_6$ [PubChem CID 9904331]	298.141	299.148	5.84	297.184, 282.141, 300.151, 301.140, 316.174, 322.133	0.610×10^8	1.32
α-Eleostearic acid [Conjugated linolenic acid]	$C_{18}H_{30}O_2$ [PubChem CID 5281115]	278.224	279.231	12.452	278.145, 278.628, 279.646, 280.148, 281.237, 282.278	0.549×10^8	1.19
Ginkgoneolic acid [Alkylphenolic acid]	$C_{20}H_{32}O_3$ [PubChem CID 161306]	320.234	321.241	10.332	303.061, 303.136, 310.163, 317.171, 321.147	0.532×10^8	1.15
Methyl palmitate [Fatty acid ester]	$C_{17}H_{34}O_2$ [PubChem CID 8181]	270.255	271.263	17.071	271.059, 271.130, 272.266, 273.220, 273.257, 273.268, 274.260, 275.200	0.486×10^8	1.05
Hirsuteine [Monoterpene indole alkaloid]	$C_{22}H_{26}N_2O_3$ [PubChem CID 3037151]	366.194	367.201	5.956	366.040, 367.048, 368.204, 369.169, 369.207, 370.163, 371.146	0.397×10^8	0.86
Sitostenone [Steroid, specifically to stigmastane and 3-oxo-delta-4-steroid]	$C_{29}H_{48}O$ [PubChem CID 5484202]	412.369	413.376	18.918	414.379, 415.383	0.377×10^8	0.82
Fagaramide [Alkylamide, specifically a type of cinnamamide and secondary carboxamide]	$C_{14}H_{17}NO_3$ [PubChem CID 5281772]	247.121	248.128	2.797	247.012, 247.997, 249.131, 249.157, 250.160, 251.088 251.164	0.342×10^8	0.74
Homovanillic acid [Methoxy phenolic carboxylic acid]	$C_9H_{10}O_4$ [PubChem CID 1738]	182.058	183.065	4.737	182.985, 183.085, 183.101, 184.068, 184.985, 185.984, 186.956, 187.126	0.323×10^8	0.70
Glycidyl oleate [Carboxylic ester and an epoxide]	$C_{21}H_{38}O_3$ [PubChem CID 5354568]	338.281	339.288	15.832	338.254, 339.343, 340.291, 341.303, 341.294, 342.307	0.301×10^8	0.65
NP-008177 [Natural product]	$C_{28}H_{41}NO_5$ [PubChem CID 4969]	471.298	472.305	12.63	471.215, 472.361, 473.308, 474.311, 475.211, 475.243, 476.373	0.295×10^8	0.64
Curcumene [Sesquiterpene]	$C_{15}H_{22}$ [PubChem CID 92139]	202.172	203.179	9.763	202.120, 203.000, 204.182, 205.085,	0.285×10^8	0.67

					205.194, 206.198,<br>207.174		
Lachnophyllum ester<br>[Polyunsaturated fatty ester]	$C_{11}H_{12}O_2$<br>[PubChem CID<br>642290	176.083	177.090	10.043	177.126, 177.163,<br>178.093, 179.069,<br>180.073, 181.064	0.271 x 10^8	0.59
Limonin<br>[Limonoid, furanolactone]	$C_{26}H_{30}O_8$<br>[PubChem CID<br>179651]	470.192	471.199	7.317	470.155, 471.797,<br>472.202, 473.142,<br>473.179, 473.204,<br>474.210, 474.228	0.264 x 10^8	0.57
NP-000615<br>[Natural product]	$C_{14}H_{12}O_4$<br>[PubChem CID<br>736489]	244.073	245.081	7.577	245.116, 245.152,<br>246.083, 247.086,<br>247.132, 248.127,<br>249.147	0.259 x 10^8	0.56
6-Pentyl-2H-pyran-2-one<br>[2-pyranones]	$C_{10}H_{14}O_2$<br>[PubChem CID<br>33960]	166.099	167.106	9.686	165.982, 167.085,<br>167.587, 168.109,<br>169.064, 170.096,<br>171.080	0259 x 10^8	0.56
(1S,4S,5R,9R,13S)-5,9-dimethyl-14-<br>methylidenetetracyclo[11.2.1.0Âa,Âaâ<br>□°.0â□´,â□a]hexadec-10-ene-5-<br>carboxylic acid<br>[Carboxylic acid]	$C_{20}H_{28}O_2$	300.208	301.216	13.83	300.288, 301.070,<br>301.140, 302.218,<br>303.231, 304.234,<br>305.246	0.241 x 10^8	0.52
Methyl isonicotinate<br>[Pyridine carboxylic acid ester]	$C_7H_7NO_2$<br>[PubChem CID<br>227085]	137.047	138.055	0.805	137.132, 139.058,<br>140.070, 140.784,<br>140.787, 141.054,<br>142.049	0.231 x 10^8	0.50
Shogaol<br>[Methoxy pheno]	$C_{17}H_{24}O_3$<br>[PubChem CID<br>5281794]	276.172	277.180	11.893	276.101, 277.137,<br>277.639, 278.638,<br>279.138, 279.156,<br>280.140, 280.160	0.227 x 10^8	0.49
4-Methoxybenzaldehyde<br>[Benzaldehyde]	$C_8H_8O_2$<br>[PubChem CID<br>31244]	136.052	137.059	10.025	137.095, 137.023,<br>137.132, 138.062,<br>139.987, 140.951	0.218 x 10^8	0.47
Lotaustralin<br>[Cyanogenic glycoside]	$C_{11}H_{19}NO_6$<br>[PubChem CID<br>441467]	261.121	262.128	0.797	264.107, 266.122,<br>274.091, 276.143,<br>277.102, 287.196	0.216 x 10^8	0.47

4-Coumaric acid [Hydroxycinnamic acid]	$C_9H_8O_3$ [PubChem CID 322]	164.047	165.055	5.203	149.059, 153.054, 158.961, 162.076, 166.057	0.215 x 10^8	0.47
Helenalin [Sesquiterpene lactone]	$C_{15}H_{18}O_4$ [PubChem CID 23205]	262.120	263.127	5.835	246.120, 246.148, 255.112, 257.149, 262.107	0.197 x 10^8	0.43
2-Amino-1,3,4-octadecanetriol [Phytosphingosine, a sphingoid base]	$C_{18}H_{39}NO_3$ [PubChem CID 10892490]	317.292	318.299	9.924	317.210, 319.225, 319.302, 320.228, 321.146, 321.240	0.196 x 10^8	0.42
Butyrin [Triglyceride]	$C_{15}H_{26}O_6$ [PubChem CID 6050]	302.176	303.179	13.2	303.191, 303.230, 304.182, 305.176, 305.210, 306.213, 307.262	0.193 x 10^8	0.42
Coumarin [Chromenone, phenolic compound]	$C_9H_6O_2$ [PubChem CID 323]	146.037	147.044	7.701	149.023, 158.961, 161.059, 163.075, 171.185, 173.132, 175.075	0.176 x 10^8	0.38
(-)-Caryophyllene oxide [Sesquiterpene]	$C_{15}H_{24}O$ [PubChem CID 1742210]	220.182	221.189	9.104	221.012, 221.049, 221.580, 222.193, 223.169, 223.988, 225.091, 225.148	0.172 x 10^8	0.37
Ethylestrenol [Pregnane steroid]	$C_{20}H_{32}O$ [PubChem CID 13765]	288.245	289.252	12.717	288.134, 288.159, 289.215, 290.255, 291.137, 293.153, 291.194, 293.210,	0.170 x 10^8	0.37
Cholest-4-en-3-one [Cholestanoid]	$C_{27}H_{44}O$ [PubChem CID 91477]	384.338	385.345	18.151	384.008, 385.936, 386.348, 387.351, 388.355, 389.268	0.160 x 10^8	0.35
Prolylleucine [Dipeptide]	$C_{11}H_{20}N_2O_3$ [PubChem CID 173815]	228.147	229.154	1.159	228.086, 229.032, 230.102, 230.157, 230.990, 231.084, 233.042, 233.063	0.155 x 10^8	0.34
Campest-4-en-3-one [Ergosterol derivative]	$C_{28}H_{46}O$ [PubChem CID 11988279]	398.354	399.361	18.499	400.364, 401.288, 401.367, 402.371	0.154 x 10^8	0.33
Eugenitin [Chromone]	$C_{12}H_{12}O_4$ [PubChem CID 3083581]	220.073	221.080	5.835	221.117, 221.153, 221.189, 222.083,	0.149 x 10^8	0.32

					223.094, 223.988, 225.148		
Hernanol [Fatty alcohol and a primary alcohol]	$C_{22}H_{26}O_7$ [PubChem CID 641789]	402.167	403.174	10.296	393.166, 399.185, 397.178, 398.181	0.147 x 10^8	0.32
Acridine-9(10H)-thione [Acridine]	$C_{13}H_9NS$ [PubChem CID 2818474]	211.045	212.052	11.728	211.122, 211.169, 213.056, 213.090, 214.048, 215.179, 216.174	0.142 x 10^8	0.31
2-[(1S)-1-Hydroxyethyl]-4(1H)-quinazolinone [Quinazolinone]	$C_{10}H_{10}N_2O_2$ [PubChem CID 135505243]	190.074	191.081	4.308	191.142, 191.179, 193.122, 192.084, 193.083, 194.081, 195.087, 195.101	0.139 x 10^8	0.30
2-{2-[5-(Ethoxycarbonyl)-2-morpholinoanilino]-2-oxoethoxy} acetic acid [Amidobenzoic acid]	$C_{17}H_{22}N_2O_7$ [PubChem CID 2811005]	366.144	367.151	9.122	367.187, 367.245, 368.154, 368.257, 369.156, 369.169, 370.173, 371.148	0.135 x 10^8	0.29
Piperine [Piperidine alkaloid]	$C_{17}H_{19}NO_3$ [PubChem CID 638024]	285.134	286.143	10.753	285.074, 286.224, 287.088, 287.236, 289.103, 289.121, 290.125	0.120 x 10^8	0.26
(4-benzyl-1,4-oxazinan-2-yl) methyl N-[(4-methylphenyl) sulfonyl] carbamate	$C_{20}H_{24}N_2O_5S$	404.140	405.148	13.227	405.261, 405.280, 406.150, 407.154, 407.276, 409.309	0.118 x 10^8	0.26
Phytosphingosine [Sphingoid lipid]	$C_{18}H_{39}NO_3$ [PubChem CID 122121]	317.292	318.299	11.949	317.209, 319.225, 319.302, 320.228, 321.147, 321.241	0.118 x 10^8	0.25
Methyl cinnamate [Cinnamic acid ester]	$C_{10}H_{10}O_2$ [PubChem CID 637520]	162.068	163.075	7.685	163.111, 163.147, 164.078, 165.075, 165.982, 167.012	0.114 x 10^8	0.25
Cinnamyl alcohol [Aromatic alcohol]	$C_9H_{10}O$ [PubChem CID 5315892]	134.073	135.080	4.963	135.116, 135.126, 136.083, 137.059, 137.132, 138.135, 139.075	0.113 x 10^8	0.24
(5R)-3-[(1S,2R,4aS,6R,8aR)-1,6-dimethyl-2-[(1E)-prop-1-en-1-yl]-1,2,4a,5,6,7,8,8a-octahydronaphthalene-1-carbonyl]-4-	$C_{22}H_{31}NO_4$	373.225	374.232	13.343	374.325, 374.362, 375.235, 375.250, 377.265, 377.319	0.112 x 10^8	0.24

hydroxy-5-(hydroxymethyl)-1-methyl-2,5-dihydro-1H-pyrrol-2-one							
Isopropyl palmitate [Fatty acid ester]	$C_{19}H_{38}O_2$ [PubChem CID 8907]	298.286	299.294	17.755	299.061, 299.090, 300.297, 301.069, 301.300, 301.140, 302.143, 303.230	0.111 x 10^8	0.24
Hexadecanamide [Fatty amide]	$C_{16}H_{33}NO$ [PubChem CID 69421]	255.256	256.263	14.45	255.174, 257.079, 257.151, 257.266, 258.278, 259.166, 259.205	0.108 x 10^8	0.23
NP-016928 [Natural product]	$C_{20}H_{28}O_3$	316.203	317.210	11.682	316.089, 317.092 318.299, 319.187 319.225, 320.229, 321.241	0.103 x 10^8	0.22
Linoleamide [Fatty amide obtained from linoleic acid]	$C_{18}H_{33}NO$ [PubChem CID 6435901]	279.256	280.263	14.025	281.266, 281.247, 282.278, 283.205 283.262	0.098 x 10^8	0.21
3',4'-Dimethoxyacetophenone [Alkyl-phenylketone]	$C_{10}H_{12}O_3$ [PubChem CID 14328]	180.078	181.085	8.36	181.121, 182.088, 183.079, 182.984, 184.561, 185.095	0.089 x 10^8	0.19
Linalyl Propionate [Acyclic monoterpenoid]	$C_{13}H_{22}O_2$ [PubChem CID 61098]	210.162	211.169	8.019	210.084, 210.933, 211.133, 212.172, 213.091, 213.148, 215.081, 215.143	0.087 x 10^8	0.19
Ethyl myristate [Fatty acid ester]	$C_{16}H_{32}O_2$ [PubChem CID 31283]	256.240	257.247	16.403	256.081, 257.151, 258.250, 259.166, 259.205, 261.221	0.083 x 10^8	0.18
Oleoyl ethanolamide [N-acylethanolamines]	$C_{20}H_{39}NO_2$ [PubChem CID 5283454]	325.297	326.304	14.047	325.272, 327.307, 329.004, 329.207, 329.246, 330.210, 330.250	0.080 x 10^8	0.17
Ethyl palmitoleate [Fatty acid ethyl esters]	$C_{18}H_{34}O_2$ [PubChem CID 6436624]	282.255	283.263	13.186	282.116, 283.650, 285.124, 285.169, 285.241, 285.220, 287.236	0.076 x 10^8	0.16
1-(4-benzhydrylpiperazino)-3-[4-(1,2,3-thiadiazol-4-yl) phenoxy]-2-propanol	$C_{28}H_{30}N_4O_2S$	486.209	487.215	8.723	488.219, 488.244, 489.226, 491.711, 491.209	0.072 x 10^8	0.15

2,2,6,6-Tetramethyl-1-piperidinol (TEMPO) [Piperidine alkaloid]	$C_9H_{19}NO$ [PubChem CID 549976]	157.146	158.154	8.886	157.064, 158.961, 159.080, 159.116, 161.059, 161.132	0.069×10^8	0.14
(+)-ar-Turmerone [Sesquiterpene]	$C_{15}H_{20}O$ [PubChem CID 160512]	216.1509	217.158	12.737	217.194, 219.173, 220.177, 221.189	0.065×10^8	0.14
